# 3,3′-Dibromo-5,5′-bis­[(*S*)-l-menth­yloxy]-4,4′-(hexane-1,6-diyldiimino)difuran-2(5*H*)-one

**DOI:** 10.1107/S1600536808020333

**Published:** 2008-07-31

**Authors:** Zhao-Yang Wang, Xiu-Mei Song, Yue-Peng Cai, Zheng-Zhou Mao

**Affiliations:** a School of Chemistry and Environment, South China Normal University, Guangzhou 510631, People’s Republic of China

## Abstract

The title compound, C_34_H_54_Br_2_N_2_O_6_, was obtained by the Michael addition–elimination reaction of (5*S*)-5-(l-menthyl­oxy)-3,4-dibromo­furan-2(5*H*)-one with 1,6-hexa­nediamine in the presence of triethyl­amine. The crystal structure contains two chiral five-membered furan­one rings, in twist and envelope conformations, and two six-membered cyclo­hexane rings in chair conformations.

## Related literature

For general background, see: Boukouvalas *et al.* (2007 ▶); Carter *et al.* (2002 ▶); Feringa & de Lange (1988 ▶); Pal *et al.* (2003 ▶).
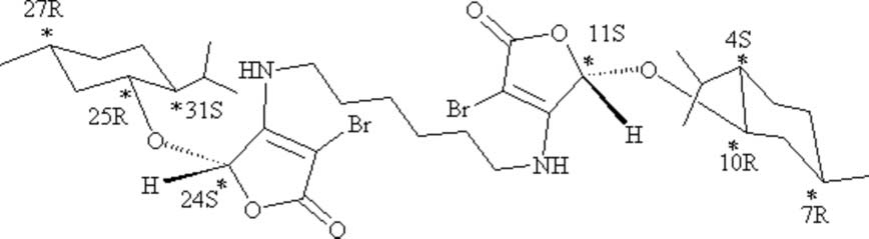


## Experimental

### 

#### Crystal data

C_34_H_54_Br_2_N_2_O_6_
*M* *_r_* = 746.61
Triclinic, 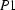

*a* = 8.2302 (2) Å
*b* = 9.1319 (2) Å
*c* = 12.7193 (3) Å
α = 105.0370 (10)°
β = 93.214 (2)°
γ = 100.499 (2)°
*V* = 902.37 (4) Å^3^
*Z* = 1
Mo *K*α radiation
μ = 2.29 mm^−1^
*T* = 298 (2) K
0.28 × 0.25 × 0.21 mm

#### Data collection

Bruker SMART CCD area-detector diffractometer
Absorption correction: multi-scan (*SADABS*; Sheldrick, 1996 ▶) *T*_min_ = 0.567, *T*_max_ = 0.645
11045 measured reflections
5935 independent reflections
4414 reflections with *I* > 2σ(*I*)
*R*_int_ = 0.032

#### Refinement

*R*[*F* ^2^ > 2σ(*F* ^2^)] = 0.038
*wR*(*F* ^2^) = 0.090
*S* = 0.97
5935 reflections
403 parameters
3 restraints
H-atom parameters constrained
Δρ_max_ = 0.50 e Å^−3^
Δρ_min_ = −0.25 e Å^−3^
Absolute structure: Flack (1983 ▶), 2587 Friedel pairs
Flack parameter: 0.001 (8)

### 

Data collection: *SMART* (Bruker, 1998 ▶); cell refinement: *SAINT* (Bruker, 1999 ▶); data reduction: *SAINT*; program(s) used to solve structure: *SHELXS97* (Sheldrick, 2008 ▶); program(s) used to refine structure: *SHELXL97* (Sheldrick, 2008 ▶); molecular graphics: *SHELXTL* (Sheldrick, 2008 ▶); software used to prepare material for publication: *SHELXTL*.

## Supplementary Material

Crystal structure: contains datablocks I, global. DOI: 10.1107/S1600536808020333/kp2170sup1.cif

Structure factors: contains datablocks I. DOI: 10.1107/S1600536808020333/kp2170Isup2.hkl

Additional supplementary materials:  crystallographic information; 3D view; checkCIF report

## Figures and Tables

**Fig. 1.**
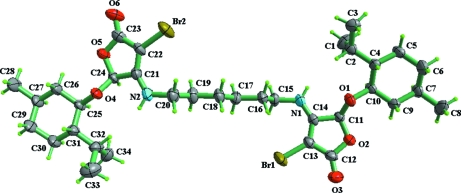
The molecular structure of the title molecule. The atom-numbering scheme is shown at the 50% probability level.

## supplementary crystallographic information

### Comment

A chiral 2(5*H*)-furanone moiety, a frequently found substructure in natural products, have received considerable attention due to the significant biological activities, such as antifungal, antibacterial, and anti-inflammatory (Pal *et al.*; 2003). It is also an important synthetic intermediates (Feringa & de Lange, 1988), and widely used in asymmetry reactions (Carter *et al.*, 2002; Boukouvalas *et al.*, 2007). Here we report the crystal structure of the title compound, *N*,*N*'-bis-[3-bromo-5-*S*-(l-menthloxy)-2(5*H*)-4-furanon-yl]-hexane- 1,6-diamine (I), namely C_34_N_2_H_54_Br_2_O_6_(Fig. 1), obtained *via* Michael addition-elimination reaction. The molecule of (I) has eight chiral centres (C4(S),C7(*R*),C10(*R*),C11(S),C24(S),C25(*R*),C27(*R*),C31(S)). The two chiral five-membered furanone rings are in a twisted conformation (C11/C14) and an envelope (C24) conformation whereas two six-membered cyclohexane rings are in the chair conformation.The bond lengths and angles in the title compound are in good agreement with expected values.

### Experimental

The title compound (I) was prepared by reaction of 5-*S*-(l-menthloxy)-3,4-dibromo-2(5*H*)-furanone with the 1,6-hexanediamine in DMF with existence of triethylamine under N_2_ atmosphere. After stirring for 4 h at room temperature, the resulting solid was isolated by silica gel column chromatography with gradient mixtures of petroleum ether and ethyl acetate. Colourless crystals of (I) were obtained by slow evaporation of a solution in acetone (yield: 78%). EIS-MS (m/*z*): 769.75 [*M*+Na]^+^ (95%). [α]_D_^25^:+52.42°.

### Refinement

The structure was solved using direct methods followed by Fourier synthesis. Non-H atoms were refined anisotropically. All of H atoms were placed in idealized positions, forced to ride on the atom to which they are bonded.

### Crystal data

**Table:**

C_34_H_54_Br_2_N_2_O_6_	*Z* = 1
*M**_r_* = 746.61	*F*_000_ = 390
Triclinic, *P*1	*D*_x_ = 1.374 Mg m^−^^3^
Hall symbol: P 1	Mo *K*α radiation λ = 0.71073 Å
*a* = 8.2302 (2) Å	Cell parameters from 2819 reflections
*b* = 9.1319 (2) Å	θ = 2.0–23.7º
*c* = 12.7193 (3) Å	µ = 2.29 mm^−^^1^
α = 105.0370 (10)º	*T* = 298 (2) K
β = 93.214 (2)º	Block, colourless
γ = 100.499 (2)º	0.28 × 0.25 × 0.21 mm
*V* = 902.37 (4) Å^3^	

### Data collection

**Table:**

Bruker SMART CCD area-detector diffractometer	5935 independent reflections
Radiation source: fine-focus sealed tube	4414 reflections with *I* > 2σ(*I*)
Monochromator: graphite	*R*_int_ = 0.032
*T* = 298(2) K	θ_max_ = 25.5º
φ and ω scans	θ_min_ = 1.7º
Absorption correction: multi-scan(SADABS; Sheldrick, 1996)	*h* = −9→9
*T*_min_ = 0.567, *T*_max_ = 0.645	*k* = −11→11
11045 measured reflections	*l* = −15→15

### Refinement

**Table:**

Refinement on *F*^2^	Hydrogen site location: inferred from neighbouring sites
Least-squares matrix: full	H-atom parameters constrained
*R*[*F*^2^ > 2σ(*F*^2^)] = 0.039	*w* = 1/[σ^2^(*F*_o_^2^) + (0.0205*P*)^2^] where *P* = (*F*_o_^2^ + 2*F*_c_^2^)/3
*wR*(*F*^2^) = 0.090	(Δ/σ)_max_ < 0.001
*S* = 0.98	Δρ_max_ = 0.50 e Å^−^^3^
5935 reflections	Δρ_min_ = −0.25 e Å^−^^3^
403 parameters	Extinction correction: none
3 restraints	Absolute structure: Flack (1983), 2587 Friedel pairs
Primary atom site location: structure-invariant direct methods	Flack parameter: 0.001 (8)
Secondary atom site location: difference Fourier map	

### Special details

**Table:**

Geometry. All e.s.d.'s (except the e.s.d. in the dihedral angle between two l.s. planes) are estimated using the full covariance matrix. The cell e.s.d.'s are taken into account individually in the estimation of e.s.d.'s in distances, angles and torsion angles; correlations between e.s.d.'s in cell parameters are only used when they are defined by crystal symmetry. An approximate (isotropic) treatment of cell e.s.d.'s is used for estimating e.s.d.'s involving l.s. planes.
Refinement. Refinement of *F*^2^ against ALL reflections. The weighted *R*-factor *wR* and goodness of fit *S* are based on *F*^2^, conventional *R*-factors *R* are based on *F*, with *F* set to zero for negative *F*^2^. The threshold expression of *F*^2^ > σ(*F*^2^) is used only for calculating *R*-factors(gt) *etc*. and is not relevant to the choice of reflections for refinement. *R*-factors based on *F*^2^ are statistically about twice as large as those based on *F*, and *R*- factors based on ALL data will be even larger.

### Fractional atomic coordinates and isotropic or equivalent isotropic displacement parameters (Å^2^)

**Table:**

	*x*	*y*	*z*	*U*_iso_*/*U*_eq_	
Br1	0.81496 (5)	0.16597 (5)	0.19540 (3)	0.0760 (2)	
Br2	−0.34832 (6)	−0.19512 (5)	−0.08054 (3)	0.0817 (2)	
O1	0.6471 (4)	0.2464 (3)	0.5844 (3)	0.0488 (9)	
O2	0.9096 (4)	0.2804 (4)	0.5257 (2)	0.0628 (8)	
O3	1.0762 (5)	0.1927 (5)	0.4010 (4)	0.0813 (12)	
O4	−0.1811 (4)	−0.2781 (3)	−0.4703 (3)	0.0416 (8)	
O5	−0.3972 (4)	−0.1676 (3)	−0.3926 (2)	0.0535 (7)	
O6	−0.5937 (5)	−0.1698 (4)	−0.2778 (3)	0.0754 (11)	
N1	0.5276 (5)	0.2891 (4)	0.3820 (3)	0.0567 (10)	
H1	0.4719	0.3169	0.4363	0.068*	
N2	−0.0273 (5)	−0.2212 (4)	−0.2574 (3)	0.0583 (10)	
H2A	0.0307	−0.2360	−0.3120	0.070*	
C1	0.3035 (7)	0.4220 (6)	0.6869 (6)	0.0815 (19)	
H1A	0.2956	0.4654	0.7633	0.122*	
H1B	0.2016	0.4191	0.6451	0.122*	
H1C	0.3938	0.4848	0.6640	0.122*	
C2	0.3345 (6)	0.2579 (5)	0.6683 (4)	0.0491 (13)	
H2	0.3483	0.2190	0.5907	0.059*	
C3	0.1811 (6)	0.1539 (6)	0.6920 (4)	0.0591 (13)	
H3A	0.2041	0.0533	0.6877	0.089*	
H3B	0.0892	0.1444	0.6390	0.089*	
H3C	0.1536	0.1987	0.7641	0.089*	
C4	0.4919 (6)	0.2472 (5)	0.7355 (4)	0.0421 (11)	
H4	0.4936	0.1367	0.7207	0.051*	
C5	0.4948 (7)	0.3092 (6)	0.8604 (4)	0.0534 (13)	
H5A	0.4883	0.4177	0.8783	0.064*	
H5B	0.3976	0.2537	0.8837	0.064*	
C6	0.6485 (9)	0.2925 (7)	0.9225 (5)	0.0574 (13)	
H6A	0.6451	0.3364	1.0003	0.069*	
H6B	0.6497	0.1834	0.9103	0.069*	
C7	0.8034 (5)	0.3711 (5)	0.8886 (3)	0.0500 (10)	
H7	0.8011	0.4816	0.9046	0.060*	
C8	0.9614 (8)	0.3565 (7)	0.9516 (5)	0.0807 (19)	
H8A	0.9573	0.3971	1.0288	0.121*	
H8B	1.0570	0.4137	0.9296	0.121*	
H8C	0.9688	0.2494	0.9359	0.121*	
C9	0.8047 (6)	0.3094 (5)	0.7646 (4)	0.0477 (11)	
H9A	0.9017	0.3668	0.7424	0.057*	
H9B	0.8141	0.2016	0.7473	0.057*	
C10	0.6510 (5)	0.3221 (5)	0.6998 (3)	0.0412 (9)	
H10	0.6504	0.4319	0.7090	0.049*	
C11	0.7495 (5)	0.3225 (5)	0.5261 (3)	0.0450 (9)	
H11	0.7621	0.4347	0.5558	0.054*	
C12	0.9450 (7)	0.2278 (6)	0.4195 (4)	0.0560 (12)	
C13	0.8051 (7)	0.2291 (5)	0.3483 (4)	0.0527 (12)	
C14	0.6817 (6)	0.2727 (5)	0.4052 (3)	0.0434 (10)	
C15	0.4440 (7)	0.2641 (5)	0.2717 (4)	0.0557 (13)	
H15A	0.5250	0.3006	0.2268	0.067*	
H15B	0.3600	0.3269	0.2771	0.067*	
C16	0.3626 (6)	0.0992 (5)	0.2142 (4)	0.0523 (12)	
H16A	0.2760	0.0628	0.2555	0.063*	
H16B	0.4442	0.0340	0.2097	0.063*	
C17	0.2882 (7)	0.0881 (6)	0.1000 (4)	0.0598 (14)	
H17A	0.2092	0.1560	0.1062	0.072*	
H17B	0.3764	0.1267	0.0605	0.072*	
C18	0.2019 (7)	−0.0710 (5)	0.0330 (4)	0.0575 (13)	
H18A	0.1150	−0.1118	0.0723	0.069*	
H18B	0.2811	−0.1388	0.0232	0.069*	
C19	0.1264 (8)	−0.0707 (6)	−0.0781 (4)	0.0631 (14)	
H19A	0.0414	−0.0088	−0.0677	0.076*	
H19B	0.2120	−0.0212	−0.1142	0.076*	
C20	0.0509 (7)	−0.2275 (6)	−0.1513 (4)	0.0580 (13)	
H20A	0.1363	−0.2885	−0.1648	0.070*	
H20B	−0.0324	−0.2790	−0.1147	0.070*	
C21	−0.1792 (6)	−0.1947 (5)	−0.2765 (3)	0.0464 (10)	
C22	−0.3149 (6)	−0.1883 (5)	−0.2240 (4)	0.0501 (12)	
C23	−0.4531 (7)	−0.1733 (5)	−0.2942 (4)	0.0543 (12)	
C24	−0.2224 (5)	−0.1659 (4)	−0.3874 (3)	0.0438 (9)	
H24	−0.1615	−0.0637	−0.3886	0.053*	
C25	−0.1587 (5)	−0.2389 (5)	−0.5739 (3)	0.0422 (9)	
H25	−0.1167	−0.1271	−0.5583	0.051*	
C26	−0.3272 (6)	−0.2820 (5)	−0.6436 (4)	0.0479 (12)	
H26A	−0.3776	−0.3886	−0.6487	0.057*	
H26B	−0.3998	−0.2169	−0.6074	0.057*	
C27	−0.3136 (6)	−0.2637 (5)	−0.7591 (3)	0.0540 (11)	
H27	−0.2764	−0.1533	−0.7531	0.065*	
C28	−0.4850 (7)	−0.3194 (8)	−0.8265 (4)	0.0748 (17)	
H28A	−0.4754	−0.3079	−0.8989	0.112*	
H28B	−0.5608	−0.2588	−0.7918	0.112*	
H28C	−0.5258	−0.4265	−0.8308	0.112*	
C29	−0.1843 (9)	−0.3484 (8)	−0.8117 (5)	0.0659 (15)	
H29A	−0.1695	−0.3286	−0.8823	0.079*	
H29B	−0.2236	−0.4589	−0.8242	0.079*	
C30	−0.0177 (7)	−0.2986 (7)	−0.7412 (4)	0.0651 (15)	
H30A	0.0262	−0.1900	−0.7341	0.078*	
H30B	0.0600	−0.3576	−0.7773	0.078*	
C31	−0.0318 (6)	−0.3223 (5)	−0.6278 (4)	0.0465 (12)	
H31	−0.0756	−0.4330	−0.6387	0.056*	
C32	0.1388 (6)	−0.2838 (6)	−0.5590 (5)	0.0545 (13)	
H32	0.1153	−0.2782	−0.4835	0.065*	
C33	0.2355 (8)	−0.4138 (7)	−0.5922 (6)	0.0851 (18)	
H33A	0.2692	−0.4188	−0.6639	0.128*	
H33B	0.1659	−0.5107	−0.5930	0.128*	
H33C	0.3320	−0.3933	−0.5406	0.128*	
C34	0.2432 (7)	−0.1293 (6)	−0.5557 (5)	0.0677 (15)	
H34A	0.2826	−0.1333	−0.6258	0.102*	
H34B	0.3362	−0.1044	−0.5007	0.102*	
H34C	0.1774	−0.0512	−0.5384	0.102*	

### Atomic displacement parameters (Å^2^)

**Table:**

	*U*^11^	*U*^22^	*U*^33^	*U*^12^	*U*^13^	*U*^23^
Br1	0.1001 (5)	0.0902 (4)	0.0445 (3)	0.0276 (3)	0.0298 (3)	0.0200 (3)
Br2	0.1127 (5)	0.0972 (4)	0.0483 (4)	0.0293 (4)	0.0368 (4)	0.0320 (3)
O1	0.058 (2)	0.0498 (18)	0.0312 (18)	0.0000 (16)	0.0030 (17)	0.0068 (14)
O2	0.0488 (19)	0.100 (2)	0.0454 (17)	0.0176 (17)	0.0113 (14)	0.0272 (16)
O3	0.064 (3)	0.117 (3)	0.085 (3)	0.040 (2)	0.030 (2)	0.047 (2)
O4	0.0459 (19)	0.0449 (17)	0.0359 (19)	0.0083 (14)	0.0140 (16)	0.0129 (14)
O5	0.0533 (19)	0.0693 (19)	0.0458 (16)	0.0212 (15)	0.0161 (14)	0.0217 (14)
O6	0.075 (3)	0.086 (3)	0.085 (3)	0.037 (2)	0.042 (2)	0.037 (2)
N1	0.063 (3)	0.070 (3)	0.0352 (19)	0.021 (2)	0.0022 (18)	0.0063 (18)
N2	0.057 (3)	0.077 (3)	0.0358 (19)	0.003 (2)	0.0035 (18)	0.0146 (18)
C1	0.062 (4)	0.066 (3)	0.119 (5)	0.013 (3)	−0.009 (3)	0.034 (3)
C2	0.052 (3)	0.047 (3)	0.045 (3)	0.005 (2)	0.006 (3)	0.011 (2)
C3	0.051 (3)	0.062 (3)	0.067 (3)	0.011 (2)	0.014 (2)	0.021 (2)
C4	0.044 (3)	0.044 (2)	0.038 (2)	0.0074 (19)	0.006 (2)	0.0116 (19)
C5	0.055 (3)	0.064 (3)	0.037 (3)	0.002 (2)	0.012 (2)	0.011 (2)
C6	0.073 (4)	0.062 (3)	0.036 (3)	0.015 (3)	0.003 (3)	0.012 (2)
C7	0.057 (3)	0.054 (3)	0.037 (2)	0.015 (2)	0.002 (2)	0.0076 (18)
C8	0.070 (4)	0.113 (5)	0.055 (4)	0.038 (3)	−0.012 (3)	0.007 (3)
C9	0.037 (3)	0.061 (3)	0.043 (3)	0.010 (2)	0.008 (2)	0.011 (2)
C10	0.045 (3)	0.041 (2)	0.0334 (19)	0.0039 (18)	0.0076 (18)	0.0065 (16)
C11	0.045 (3)	0.050 (2)	0.041 (2)	0.0070 (19)	0.0072 (19)	0.0140 (18)
C12	0.056 (3)	0.068 (3)	0.055 (3)	0.022 (3)	0.024 (3)	0.028 (2)
C13	0.063 (3)	0.059 (3)	0.042 (2)	0.015 (2)	0.013 (2)	0.021 (2)
C14	0.054 (3)	0.041 (2)	0.036 (2)	0.008 (2)	0.007 (2)	0.0124 (17)
C15	0.066 (3)	0.062 (3)	0.039 (3)	0.019 (2)	−0.003 (2)	0.011 (2)
C16	0.054 (3)	0.068 (3)	0.037 (3)	0.013 (2)	0.005 (2)	0.019 (2)
C17	0.053 (3)	0.072 (3)	0.050 (3)	0.001 (2)	−0.002 (2)	0.018 (2)
C18	0.070 (4)	0.068 (3)	0.036 (3)	0.012 (3)	0.004 (3)	0.020 (2)
C19	0.078 (4)	0.063 (3)	0.046 (3)	0.006 (3)	−0.003 (3)	0.019 (2)
C20	0.064 (3)	0.064 (3)	0.047 (3)	0.009 (2)	0.001 (2)	0.021 (2)
C21	0.052 (3)	0.046 (2)	0.036 (2)	−0.001 (2)	0.004 (2)	0.0097 (18)
C22	0.064 (3)	0.053 (3)	0.034 (2)	0.010 (2)	0.018 (2)	0.0109 (19)
C23	0.072 (4)	0.043 (3)	0.053 (3)	0.017 (2)	0.025 (3)	0.015 (2)
C24	0.050 (3)	0.044 (2)	0.034 (2)	0.0030 (18)	0.0106 (18)	0.0090 (16)
C25	0.047 (3)	0.049 (2)	0.033 (2)	0.0101 (19)	0.0094 (18)	0.0146 (17)
C26	0.053 (3)	0.056 (3)	0.036 (2)	0.012 (2)	0.011 (2)	0.0127 (19)
C27	0.059 (3)	0.067 (3)	0.037 (2)	0.009 (2)	0.009 (2)	0.019 (2)
C28	0.068 (4)	0.124 (5)	0.037 (3)	0.022 (3)	0.005 (3)	0.029 (3)
C29	0.065 (4)	0.091 (4)	0.037 (3)	0.007 (3)	0.017 (3)	0.014 (3)
C30	0.061 (4)	0.087 (4)	0.042 (3)	0.012 (3)	0.016 (3)	0.010 (2)
C31	0.042 (3)	0.047 (3)	0.050 (3)	0.007 (2)	0.015 (2)	0.011 (2)
C32	0.044 (3)	0.071 (3)	0.057 (3)	0.017 (2)	0.015 (3)	0.028 (3)
C33	0.070 (4)	0.072 (4)	0.125 (5)	0.021 (3)	0.020 (4)	0.043 (3)
C34	0.056 (3)	0.064 (3)	0.074 (4)	0.015 (2)	−0.009 (3)	0.005 (3)

### Geometric parameters (Å, °)

**Table:**

Br1—C13	1.890 (5)	C15—C16	1.502 (7)
Br2—C22	1.876 (5)	C15—H15A	0.9700
O1—C11	1.366 (5)	C15—H15B	0.9700
O1—C10	1.448 (5)	C16—C17	1.516 (7)
O2—C12	1.378 (6)	C16—H16A	0.9700
O2—C11	1.438 (5)	C16—H16B	0.9700
O3—C12	1.200 (6)	C17—C18	1.502 (6)
O4—C24	1.373 (5)	C17—H17A	0.9700
O4—C25	1.464 (5)	C17—H17B	0.9700
O5—C23	1.368 (5)	C18—C19	1.512 (6)
O5—C24	1.433 (5)	C18—H18A	0.9700
O6—C23	1.192 (6)	C18—H18B	0.9700
N1—C14	1.330 (5)	C19—C20	1.491 (7)
N1—C15	1.471 (6)	C19—H19A	0.9700
N1—H1	0.8600	C19—H19B	0.9700
N2—C21	1.337 (6)	C20—H20A	0.9700
N2—C20	1.482 (6)	C20—H20B	0.9700
N2—H2A	0.8600	C21—C22	1.335 (7)
C1—C2	1.526 (7)	C21—C24	1.535 (5)
C1—H1A	0.9600	C22—C23	1.454 (7)
C1—H1B	0.9600	C24—H24	0.9800
C1—H1C	0.9600	C25—C31	1.497 (6)
C2—C3	1.530 (7)	C25—C26	1.532 (6)
C2—C4	1.544 (7)	C25—H25	0.9800
C2—H2	0.9800	C26—C27	1.529 (6)
C3—H3A	0.9600	C26—H26A	0.9700
C3—H3B	0.9600	C26—H26B	0.9700
C3—H3C	0.9600	C27—C29	1.513 (9)
C4—C10	1.513 (6)	C27—C28	1.536 (7)
C4—C5	1.539 (7)	C27—H27	0.9800
C4—H4	0.9800	C28—H28A	0.9600
C5—C6	1.506 (9)	C28—H28B	0.9600
C5—H5A	0.9700	C28—H28C	0.9600
C5—H5B	0.9700	C29—C30	1.521 (9)
C6—C7	1.489 (8)	C29—H29A	0.9700
C6—H6A	0.9700	C29—H29B	0.9700
C6—H6B	0.9700	C30—C31	1.521 (7)
C7—C9	1.533 (6)	C30—H30A	0.9700
C7—C8	1.533 (7)	C30—H30B	0.9700
C7—H7	0.9800	C31—C32	1.544 (7)
C8—H8A	0.9600	C31—H31	0.9800
C8—H8B	0.9600	C32—C34	1.502 (7)
C8—H8C	0.9600	C32—C33	1.530 (7)
C9—C10	1.509 (6)	C32—H32	0.9800
C9—H9A	0.9700	C33—H33A	0.9600
C9—H9B	0.9700	C33—H33B	0.9600
C10—H10	0.9800	C33—H33C	0.9600
C11—C14	1.526 (5)	C34—H34A	0.9600
C11—H11	0.9800	C34—H34B	0.9600
C12—C13	1.429 (7)	C34—H34C	0.9600
C13—C14	1.339 (7)		
			
C11—O1—C10	116.8 (3)	C18—C17—H17A	108.3
C12—O2—C11	109.9 (3)	C16—C17—H17A	108.3
C24—O4—C25	116.0 (3)	C18—C17—H17B	108.3
C23—O5—C24	109.9 (3)	C16—C17—H17B	108.3
C14—N1—C15	125.9 (4)	H17A—C17—H17B	107.4
C14—N1—H1	117.1	C17—C18—C19	112.2 (3)
C15—N1—H1	117.1	C17—C18—H18A	109.2
C21—N2—C20	126.3 (4)	C19—C18—H18A	109.2
C21—N2—H2A	116.9	C17—C18—H18B	109.2
C20—N2—H2A	116.9	C19—C18—H18B	109.2
C2—C1—H1A	109.5	H18A—C18—H18B	107.9
C2—C1—H1B	109.5	C20—C19—C18	114.4 (4)
H1A—C1—H1B	109.5	C20—C19—H19A	108.7
C2—C1—H1C	109.5	C18—C19—H19A	108.7
H1A—C1—H1C	109.5	C20—C19—H19B	108.7
H1B—C1—H1C	109.5	C18—C19—H19B	108.7
C1—C2—C3	109.2 (5)	H19A—C19—H19B	107.6
C1—C2—C4	114.2 (4)	N2—C20—C19	112.5 (4)
C3—C2—C4	110.5 (4)	N2—C20—H20A	109.1
C1—C2—H2	107.6	C19—C20—H20A	109.1
C3—C2—H2	107.6	N2—C20—H20B	109.1
C4—C2—H2	107.6	C19—C20—H20B	109.1
C2—C3—H3A	109.5	H20A—C20—H20B	107.8
C2—C3—H3B	109.5	C22—C21—N2	136.9 (4)
H3A—C3—H3B	109.5	C22—C21—C24	106.2 (4)
C2—C3—H3C	109.5	N2—C21—C24	116.9 (4)
H3A—C3—H3C	109.5	C21—C22—C23	111.1 (4)
H3B—C3—H3C	109.5	C21—C22—Br2	130.3 (4)
C10—C4—C5	109.4 (4)	C23—C22—Br2	118.6 (4)
C10—C4—C2	112.9 (4)	O6—C23—O5	121.9 (5)
C5—C4—C2	114.8 (4)	O6—C23—C22	130.5 (5)
C10—C4—H4	106.4	O5—C23—C22	107.5 (4)
C5—C4—H4	106.4	O4—C24—O5	113.3 (3)
C2—C4—H4	106.4	O4—C24—C21	109.9 (3)
C6—C5—C4	112.9 (5)	O5—C24—C21	104.5 (3)
C6—C5—H5A	109.0	O4—C24—H24	109.7
C4—C5—H5A	109.0	O5—C24—H24	109.7
C6—C5—H5B	109.0	C21—C24—H24	109.7
C4—C5—H5B	109.0	O4—C25—C31	107.8 (3)
H5A—C5—H5B	107.8	O4—C25—C26	109.0 (3)
C7—C6—C5	112.1 (5)	C31—C25—C26	113.2 (4)
C7—C6—H6A	109.2	O4—C25—H25	108.9
C5—C6—H6A	109.2	C31—C25—H25	108.9
C7—C6—H6B	109.2	C26—C25—H25	108.9
C5—C6—H6B	109.2	C27—C26—C25	112.9 (4)
H6A—C6—H6B	107.9	C27—C26—H26A	109.0
C6—C7—C9	109.5 (4)	C25—C26—H26A	109.0
C6—C7—C8	112.9 (4)	C27—C26—H26B	109.0
C9—C7—C8	111.6 (4)	C25—C26—H26B	109.0
C6—C7—H7	107.5	H26A—C26—H26B	107.8
C9—C7—H7	107.5	C29—C27—C26	109.8 (4)
C8—C7—H7	107.5	C29—C27—C28	112.7 (4)
C7—C8—H8A	109.5	C26—C27—C28	109.9 (4)
C7—C8—H8B	109.5	C29—C27—H27	108.1
H8A—C8—H8B	109.5	C26—C27—H27	108.1
C7—C8—H8C	109.5	C28—C27—H27	108.1
H8A—C8—H8C	109.5	C27—C28—H28A	109.5
H8B—C8—H8C	109.5	C27—C28—H28B	109.5
C10—C9—C7	113.1 (4)	H28A—C28—H28B	109.5
C10—C9—H9A	109.0	C27—C28—H28C	109.5
C7—C9—H9A	109.0	H28A—C28—H28C	109.5
C10—C9—H9B	109.0	H28B—C28—H28C	109.5
C7—C9—H9B	109.0	C27—C29—C30	112.2 (5)
H9A—C9—H9B	107.8	C27—C29—H29A	109.2
O1—C10—C9	111.5 (4)	C30—C29—H29A	109.2
O1—C10—C4	106.1 (3)	C27—C29—H29B	109.2
C9—C10—C4	112.8 (3)	C30—C29—H29B	109.2
O1—C10—H10	108.8	H29A—C29—H29B	107.9
C9—C10—H10	108.8	C29—C30—C31	112.2 (5)
C4—C10—H10	108.8	C29—C30—H30A	109.2
O1—C11—O2	111.5 (3)	C31—C30—H30A	109.2
O1—C11—C14	110.6 (3)	C29—C30—H30B	109.2
O2—C11—C14	103.8 (3)	C31—C30—H30B	109.2
O1—C11—H11	110.2	H30A—C30—H30B	107.9
O2—C11—H11	110.2	C25—C31—C30	109.6 (4)
C14—C11—H11	110.2	C25—C31—C32	114.7 (4)
O3—C12—O2	120.6 (5)	C30—C31—C32	112.5 (4)
O3—C12—C13	131.6 (5)	C25—C31—H31	106.5
O2—C12—C13	107.7 (4)	C30—C31—H31	106.5
C14—C13—C12	111.1 (4)	C32—C31—H31	106.5
C14—C13—Br1	130.3 (4)	C34—C32—C33	111.5 (5)
C12—C13—Br1	118.5 (4)	C34—C32—C31	114.3 (4)
N1—C14—C13	136.4 (4)	C33—C32—C31	112.0 (5)
N1—C14—C11	116.7 (4)	C34—C32—H32	106.1
C13—C14—C11	106.8 (4)	C33—C32—H32	106.1
N1—C15—C16	115.2 (4)	C31—C32—H32	106.1
N1—C15—H15A	108.5	C32—C33—H33A	109.5
C16—C15—H15A	108.5	C32—C33—H33B	109.5
N1—C15—H15B	108.5	H33A—C33—H33B	109.5
C16—C15—H15B	108.5	C32—C33—H33C	109.5
H15A—C15—H15B	107.5	H33A—C33—H33C	109.5
C15—C16—C17	110.0 (4)	H33B—C33—H33C	109.5
C15—C16—H16A	109.7	C32—C34—H34A	109.5
C17—C16—H16A	109.7	C32—C34—H34B	109.5
C15—C16—H16B	109.7	H34A—C34—H34B	109.5
C17—C16—H16B	109.7	C32—C34—H34C	109.5
H16A—C16—H16B	108.2	H34A—C34—H34C	109.5
C18—C17—C16	115.7 (4)	H34B—C34—H34C	109.5
			
C1—C2—C4—C10	−67.7 (6)	C17—C18—C19—C20	175.3 (5)
C3—C2—C4—C10	168.8 (4)	C21—N2—C20—C19	−82.3 (6)
C1—C2—C4—C5	58.7 (6)	C18—C19—C20—N2	177.8 (5)
C3—C2—C4—C5	−64.9 (5)	C20—N2—C21—C22	−11.8 (8)
C10—C4—C5—C6	−53.3 (5)	C20—N2—C21—C24	169.8 (4)
C2—C4—C5—C6	178.5 (4)	N2—C21—C22—C23	−172.6 (5)
C4—C5—C6—C7	57.0 (6)	C24—C21—C22—C23	5.8 (5)
C5—C6—C7—C9	−55.4 (6)	N2—C21—C22—Br2	7.6 (9)
C5—C6—C7—C8	179.6 (5)	C24—C21—C22—Br2	−173.9 (3)
C6—C7—C9—C10	54.3 (5)	C24—O5—C23—O6	177.1 (4)
C8—C7—C9—C10	−179.9 (4)	C24—O5—C23—C22	−4.6 (4)
C11—O1—C10—C9	−75.9 (5)	C21—C22—C23—O6	177.0 (5)
C11—O1—C10—C4	160.9 (3)	Br2—C22—C23—O6	−3.2 (7)
C7—C9—C10—O1	−173.2 (3)	C21—C22—C23—O5	−1.1 (5)
C7—C9—C10—C4	−53.9 (5)	Br2—C22—C23—O5	178.7 (3)
C5—C4—C10—O1	174.0 (4)	C25—O4—C24—O5	84.4 (4)
C2—C4—C10—O1	−56.8 (5)	C25—O4—C24—C21	−159.1 (3)
C5—C4—C10—C9	51.6 (5)	C23—O5—C24—O4	127.4 (3)
C2—C4—C10—C9	−179.2 (4)	C23—O5—C24—C21	7.8 (4)
C10—O1—C11—O2	90.3 (4)	C22—C21—C24—O4	−130.1 (4)
C10—O1—C11—C14	−154.7 (3)	N2—C21—C24—O4	48.7 (5)
C12—O2—C11—O1	124.9 (4)	C22—C21—C24—O5	−8.3 (4)
C12—O2—C11—C14	5.8 (4)	N2—C21—C24—O5	170.5 (3)
C11—O2—C12—O3	176.9 (5)	C24—O4—C25—C31	149.2 (3)
C11—O2—C12—C13	−2.2 (5)	C24—O4—C25—C26	−87.5 (4)
O3—C12—C13—C14	178.0 (6)	O4—C25—C26—C27	−173.0 (3)
O2—C12—C13—C14	−3.0 (5)	C31—C25—C26—C27	−53.1 (5)
O3—C12—C13—Br1	0.0 (8)	C25—C26—C27—C29	51.6 (5)
O2—C12—C13—Br1	179.0 (3)	C25—C26—C27—C28	176.1 (4)
C15—N1—C14—C13	−2.3 (8)	C26—C27—C29—C30	−53.9 (6)
C15—N1—C14—C11	173.3 (4)	C28—C27—C29—C30	−176.8 (5)
C12—C13—C14—N1	−177.6 (5)	C27—C29—C30—C31	57.4 (7)
Br1—C13—C14—N1	0.1 (9)	O4—C25—C31—C30	174.1 (3)
C12—C13—C14—C11	6.5 (5)	C26—C25—C31—C30	53.4 (5)
Br1—C13—C14—C11	−175.8 (3)	O4—C25—C31—C32	−58.3 (5)
O1—C11—C14—N1	55.9 (5)	C26—C25—C31—C32	−179.0 (4)
O2—C11—C14—N1	175.7 (4)	C29—C30—C31—C25	−55.6 (6)
O1—C11—C14—C13	−127.3 (4)	C29—C30—C31—C32	175.6 (5)
O2—C11—C14—C13	−7.5 (4)	C25—C31—C32—C34	−76.0 (6)
C14—N1—C15—C16	84.6 (6)	C30—C31—C32—C34	50.1 (6)
N1—C15—C16—C17	−177.4 (4)	C25—C31—C32—C33	156.0 (5)
C15—C16—C17—C18	−179.8 (4)	C30—C31—C32—C33	−77.9 (6)
C16—C17—C18—C19	178.0 (6)		
